# Laparoscopic Transcystic Common Bile Duct Exploration: Advantages over Laparoscopic Choledochotomy

**DOI:** 10.1371/journal.pone.0162885

**Published:** 2016-09-26

**Authors:** Qian Feng, Yong Huang, Kai Wang, Rongfa Yuan, Xiaoli Xiong, Linquan Wu

**Affiliations:** 1 Department of General Surgery, The Second Affiliated Hospital of Nanchang University, Nanchang, 330006, China; 2 Department of Radiology, The Second Affiliated Hospital of Nanchang University, Nanchang, 330006, China; Digestive Disease Research Center, Scott & White Healthcare, UNITED STATES

## Abstract

**Purpose:**

The ideal treatment for choledocholithiasis should be simple, readily available, reliable, minimally invasive and cost-effective for patients. We performed this study to compare the benefits and drawbacks of different laparoscopic approaches (transcystic and choledochotomy) for removal of common bile duct stones.

**Methods:**

A systematic search was implemented for relevant literature using Cochrane, PubMed, Ovid Medline, EMBASE and Wanfang databases. Both the fixed-effects and random-effects models were used to calculate the odds ratio (OR) or the mean difference (MD) with 95% confidence interval (CI) for this study.

**Results:**

The meta-analysis included 18 trials involving 2,782 patients. There were no statistically significant differences between laparoscopic choledochotomy for common bile duct exploration (LCCBDE) (*n* = 1,222) and laparoscopic transcystic common bile duct exploration (LTCBDE) (*n* = 1,560) regarding stone clearance (OR 0.73, 95% CI 0.50–1.07; *P* = 0.11), conversion to other procedures (OR 0.62, 95% CI 0.21–1.79; *P* = 0.38), total morbidity (OR 1.65, 95% CI 0.92–2.96; *P* = 0.09), operative time (MD 12.34, 95% CI −0.10–24.78; *P* = 0.05), and blood loss (MD 1.95, 95% CI −9.56–13.46; *P* = 0.74). However, the LTCBDE group showed significantly better results for biliary morbidity (OR 4.25, 95% CI 2.30–7.85; *P*<0.001), hospital stay (MD 2.52, 95% CI 1.29–3.75; *P*<0.001), and hospital expenses (MD 0.30, 95% CI 0.23–0.37; *P*<0.001) than the LCCBDE group.

**Conclusions:**

LTCBDE is safer than LCCBDE, and is the ideal treatment for common bile duct stones.

## Introduction

Approximately 10% of gallstone patients have concomitant common bile duct (CBD) stones [1, 2], which are related to serious complications such as cholangitis and pancreatitis. Therefore, it is particularly important to improve and standardize the process of diagnosis and treatment of CBD stones. The conventional approach of open CBD exploration is considered an effective treatment option [3–5]. However, surgical trauma, bile leakage, biliary tract blood loss, and other complications are not conducive to postoperative rehabilitation [6]. With rapid developments in technology, laparoscopic CBD exploration (LCBDE) was proven to be safe, cost-effective, and reliable, regardless of whether it was performed as elective or emergency treatment [7, 8]. Clinical prospective randomized trials have shown that laparoscopic procedures are superior to open operations with regard to reduced postoperative hospital stay, morbidity and postsurgical pain [9, 10].

Laparoscopic surgery for CBD stones could be categorized into transcystic and choledochotomy approaches [11]. In laparoscopic choledochotomy for CBD exploration (LCCBDE), the integrity of the CBD is lost due to the incision over the duct. In contrast, the cystic duct is used for laparoscopic transcystic CBD exploration (LTCBDE), thus minimizing the size of the incision over the CBD [12]. Moreover, application of a separate laparoscopic suture over the stump of the cystic duct dramatically decreases the postoperative incidence of bile leakage [6]. Our research and that of several groups has reported that LTCBDE is certainly the least invasive, safest, and most efficient option, with low morbidity rates [13–15]. LTCBDE avoids choledochotomy and eliminates the subsequent requirement of a T-tube; thus avoiding T-tube prolapse or displacement, CBD stricture, electrolyte imbalance, acid-base balance disorders, and complications caused by bile drainage and invasive treatments [16]. Additionally, the treatment facilitates rapid recovery. In LCCBDE, the traditional approach is to incise the common bile duct from the front. The operative procedure is simpler than LTCBDE, and it is safe and mature.

LTCBDE has not been universally accepted because the operation is relatively complex [17], and multi-center studies have not been conducted. Furthermore, no clear guidelines for the indications of the transcystic approach versus choledochotomy are available. The aim of this study was to pool analysis by investigating published data on LCBDE by the transcystic approach and choledochotomy. We assumed that LTCBDE is the ideal treatment for CBD stones.

## Methods

### Literature search

A systematic search was carried out using Cochrane, PubMed, Ovid Medline, EMBASE and Wanfang databases using the following keywords: bile duct exploration, laparoscopic CBD exploration, laparoscopic cholecystectomy, CBD stones, LCBDE, choledochotomy, laparoscopic transcystic CBD exploration, or LTCBDE. The latest search was updated on May 20th, 2016. The eligible studies were independently selected by two reviewers. Disagreement on article inclusion between the two reviewers was resolved by discussion with a third reviewer.

### Inclusion and exclusion criteria

All controlled experimental studies about LTCBDE + laparoscopic cholecystectomy (LC) compared with LCCBDE+ LC were selected. The following were the inclusion criteria for the selected studies: (1) Patients with no contraindications for the laparoscopic approach, with no requirement of additional procedures; (2) Patients with confirmed or suspected CBD stones with gallstones; (3) Studies that included data on stone clearance, conversion to other procedures, total and biliary morbidity, blood loss (ml), length of hospital stay (days), operative time (min) and hospital expenses (wan renminbi).

The total morbidity (bile leakage, biliary stricture, bleeding, clinical pancreatitis, pneumonia, acute myocardial infarction, cholangitis, sepsis, cerebrovascular events, early reoperation, and pulmonary embolus), bile duct clearance and biliary morbidity (bile leakage and biliary stricture), conversion to other procedures (any endoscopic or surgical procedure other than the one allocated for failed bile duct clearance or any procedure for the management of a complication), operative time, length of hospital stay, hospital expenses, and blood loss were included.

The exclusion criteria were non-experimental trials, articles not reporting outcomes, editorials, review articles, and nonhuman studies. The names of the authors and the journals containing the literature did not influence our decision on the articles.

### Statistical analysis

The analyses were performed using Review Manager version 5.1 (RevMan, Cochrane Collaboration, Oxford, England). The results of this study were expressed as the odds ratios (ORs) for dichotomous data and mean difference (MD) for continuous data, with 95% confidence intervals (CIs) for both. The inverse variance method was used for continuous variables, while the Mantel-Haenzsel method was used for dichotomous variables. Statistical heterogeneity was evaluated by χ^2^ test. *P* < 0.05 was considered significant. If heterogeneity was significant, we used the random-effects model. Otherwise, we used the fixed-effects model. If data reported a median and range rather than a mean and standard deviation (SD), then the mean and SD were estimated as described previously [18].

## Results

### Study selection and characteristics

**Fig 1:**
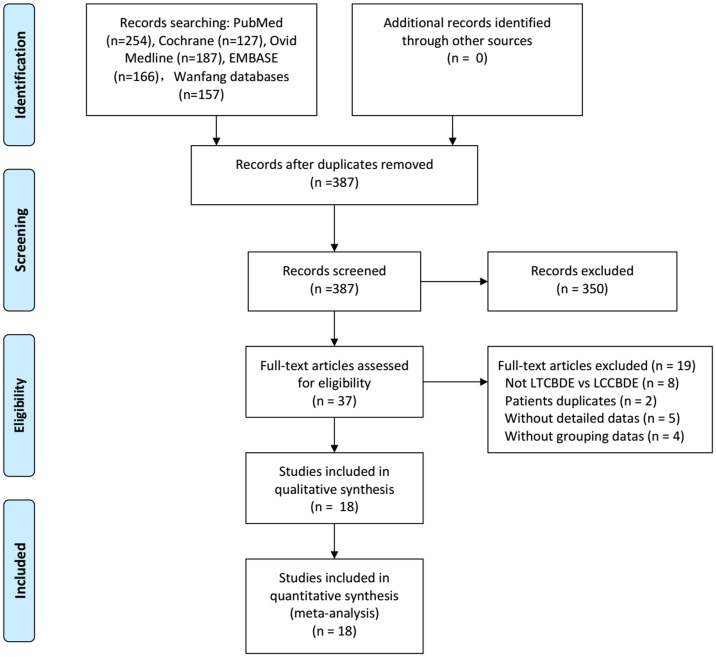
Flow diagram of study selection.

Fig 1 illustrates the final selection of relevant studies and our search process. We analyzed 18 trials [19–36] that met the criteria, involving 2,782 patients (Table 1).

**Table 1:** Description of included trials.

Author	Year	LCCBDE/LTCBDE	Conversion to other procedure	Stone clearance	Total morbidity	Biliary morbidity	Operative time (min)	Hospital stay (day)	Hospital expenses (WanRMB)	Blood loss (ml)
Martin [12]	1998	55/158	-	54/145	3/14	2/0	136.25±59.53/126.25±78.31^a^	3.25±2.15/4.5±3.75 ^a^	-	-
Rhodes [13]	1998	12/28	5/5	7/23	0/6	-	-	-	-	-
Cuschieri [14]	1999	53/56	8/11	45/45	-	-	-	-	-	-
Tokumura [15]	2002	126/91	1/13	123/89	12/19	7/1	159±42/131±35	15.6±8.7/8.1±2.4	-	-
Waage [16]	2003	57/118	5/8	55/112	-	-	260±148.87/251±87.3 ^a^	6.4±5.6/7.6±6.1 ^a^	-	-
Topal [17]	2007	30/83	2/2	28/77	5/4	-	115±54.35/105±63.05 ^a^	12±10.2/7±7.51 ^a^	-	-
Chen[18]	2007	24/40	0/0	24/40	0/0	0/0	-	9.8±1.9/7.8±1.3	1.076±0.124/0.869±0.109	34±10/26±7
Paganini[19]	2007	138/191	-	126/185	29/25	3/2	-	-	-	-
Jameel [20]	2008	50/9	-	-	9/0	-	94.21±21.67/112.86±19.69 ^a^	7.33±5.78/5.72±3.54 ^a^		-
ElGeidie[21]	2011	49/57	2/0	47/56	8/4	5/3	-	3.8±2.41/1.6±0.62 ^a^	-	-
Grubnik [22]	2012	62/76	1/1	58/72	-	-	96.25±33.39/71±23.67 ^a^	7.6±2.5/3.4±1.7	-	-
Chen [23]	2013	100/110	-	-	10/1	-	120±42.2/100±30.4	7.9±1/3.6±0.9	-	-
Tao[24]	2013	59/59	0/13	1/13	10/2	9/2	81.8±18.6/85.5±20.9	-	-	-
Poh [25]	2014	3/80	-	3/44	-	-	-	6.5±0.65/6±1.74 ^a^	-	-
Wu[26]	2014	33/29	-	33/29	-	4/0	107.19±14.58/115.68±16.64	5.1±0.6/4.43±0.38	1.1197±0.0794/0.7944±0.0833	15.5±4.6/19.25±4.3
Zhang [27]	2015	93/237	5/11	89/228	24/32	7/3	116.1±28.1/76±20.2	6.7±2.8/3.9±1.8	1.09687±0.11564/0.74353±0.09948	-
Aawsaj[28]	2015	233/85	0/12	-	-	14/0	144±48.67/107.5±52.96 ^a^	10.5±7.4/2.75±2.0 7 ^a^	-	-
Huang[29]	2015	45/53	-	-	7/8	1/2	112.41±19.63/115.36±21.51	8.32±1.15/8.15±0.35	-	-

LCCBDE, laparoscopic choledochotomy for common bile duct exploration; LTCBDE, laparoscopic transcystic common bile duct exploration;

^a^ The mean and SD were estimated by median and a range.

### CBD Stone clearance

**Fig 2:**
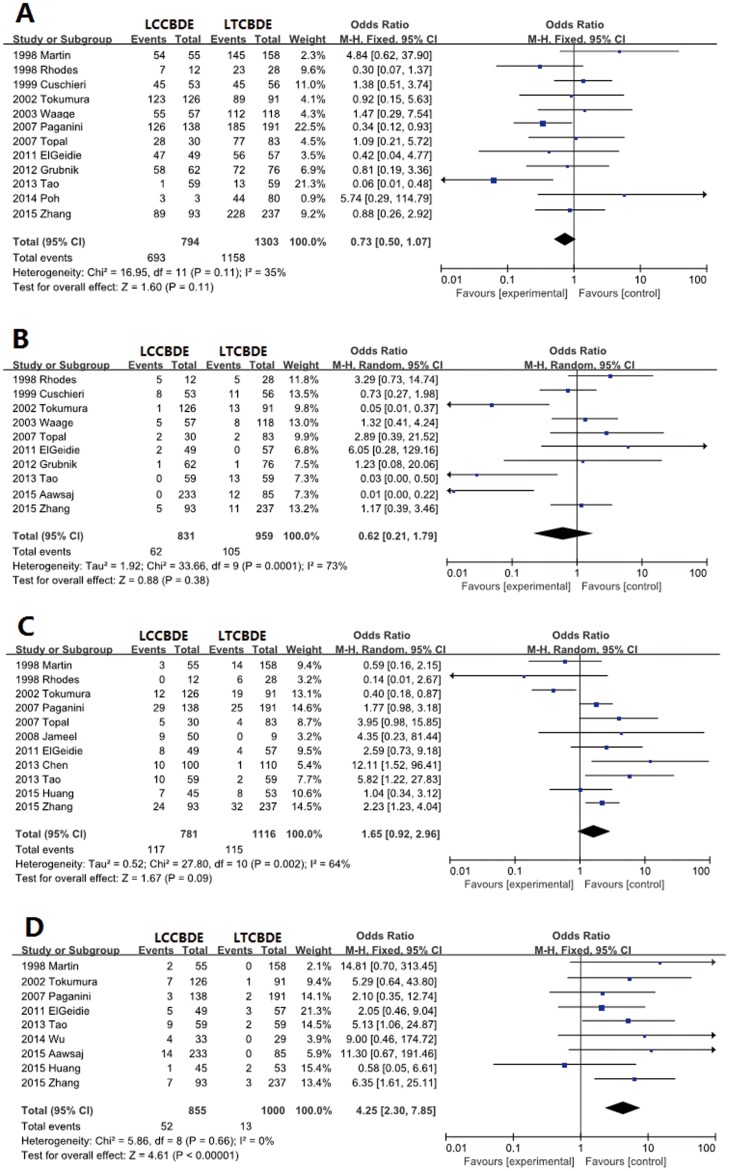
Forest plot of meta-analysis. Fixed-effect models of odds ratio for stone clearance (A), random-effects model of odds ratio for conversion to other procedures (B), random-effects model of odds ratio for total morbidity, (C) and fixed-effects model of odds ratio for biliary morbidity (D).

Stone clearance was reported in 12 trials. Stone clearance from the CBD was achieved in 87.3% of patients (693 of 794) in the LCCBDE group and in 88.9% (1,158 of 1,303) in the LTCBDE group. There was no significant difference between the two groups (OR 0.73, 95% CI 0.50–1.07; *P* = 0.11) (Fig 2A).

### Conversion

We identified ten trials with relevant data. Conversion occurred in 7.5% (62 of 831) and 10.9% (105 of 959) of patients in the LCCBDE and LTCBDE groups, respectively. There was also no statistically significant difference between the two groups (OR 0.62, 95% CI 0.21–1.79; *P* = 0.38) (Fig 2B).

### Total morbidity

Eleven trials reported total morbidity, with rates of 15.0% (117 of 781) and 10.3% (115 of 1,116) in the LCCBDE and LTCBDE groups, respectively. There was also no significant difference between the two groups (OR 1.65, 95% CI 0.92–2.96; *P* = 0.09) (Fig 2C).

### Biliary morbidity

Biliary morbidity was reported in nine trials, and occurred in 6.1% (52 of 855) of patients in the LCCBDE group versus 1.3% (13 of 1,000) in the LTCBDE group. The biliary morbidity for LTCBDE was significantly lower than in the LCCBDE group (OR 4.25, 95% CI 2.30–7.85; *P*<0.001). (Fig 2D).

### Operative time

**Fig 3:**
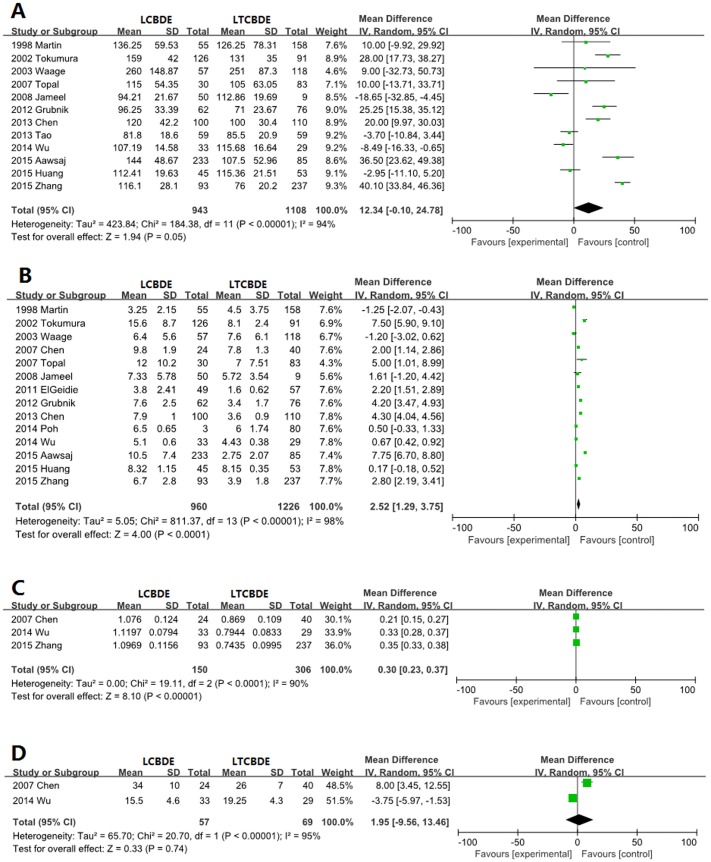
Forest plot of meta-analysis. Random effect models of mean difference for operative time (A), length of hospital stay (B), hospital expenses (C) and blood loss (D).

Twelve trials included data about for operative time. There was still no significant difference between the two groups (MD 12.34, 95% CI −0.10 to 24.78; *P* = 0.05) (Fig 3A).

### Length of hospital stay

The length of hospital stay was evaluated in 14 studies. Consequently, according to our predefined plan, the median and range were converted to mean and SD as described previously [18]. The length of hospital stay of the LTCBDE group was 2.52 days shorter than that of the LCCBDE group (MD 2.52 days, 95% CI 1.29–3.75; *P*<0.001) (Fig 3B).

### Hospitalization expenses

The hospitalization charges were recorded in only three trials. The hospital expenses in the LTCBDE group were significantly lower than in the LCCBDE group (MD 0.30 WanRMB, 95% CI 0.23–0.37; *P*<0.001) (Fig 3C).

### Blood loss

Only two trials included information about blood loss. There was still no significant difference between the two groups (MD 1.95 ml, 95% CI −9.56 to 13.46; *P* = 0.74) (Fig 3D).

### Publication bias

**Fig 4:**
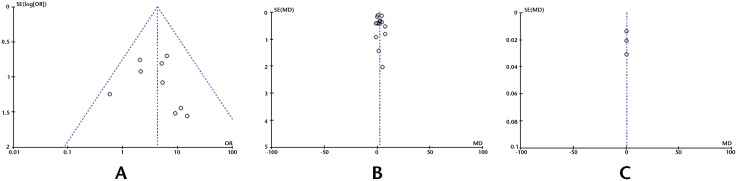
Funnel plots for meta-analysis. A, Nine articles in the meta-analysis of biliary morbidity; B, 14 articles in the meta-analysis of length of hospital stay. C, Three articles in the meta-analysis of hospital expenses.

In this meta-analysis, the funnel plot shapes for postoperative complications and postoperative biliary complications showed basic symmetry (Fig 4). No significant publication bias was observed. The results were similar and the combined results were highly reliable.

## Discussion

Currently, there are three options for treatment of CBD stones: (1) simultaneous classic CBD exploration and cholecystectomy using the laparotomy approach, (2) combined endoscopic and LC treatment in a two-step procedure, or (3) simultaneous cholecystectomy and CBD exploration using the laparoscopic approach. Surgeons should be familiar with the limitations and benefits of all these treatment strategies. We performed this study to compare the benefits and drawbacks of different laparoscopic approaches (transcystic and choledochotomy) for removal of CBD stones. We found that LTCBDE is safer than LCCBDE, and is the ideal treatment for CBD stones.

Endoscopic choledocholithiasis surgery is a mature technology; however, it is associated with high complication (19%), failure (10–15%), and mortality (3%) rates [37]. It can not only lead to postoperative complications such as pancreatitis, perforation, blood loss, sepsis, and death, but also to disruption of the sphincter of Oddi, thus causing injury to the physiological barrier function of the sphincter that prevents cholangitis due to duodenobiliary reflux [38, 39]. Natsui et al. [40] reported that the incidence of biliary bacterial contamination 30 months after endoscopic sphincterotomy (EST) was about 78%. Another study reported that the postoperative acute cholangitis rate was 2.4–10.3% in EST [41]. The randomized trials [42] and meta-analysis [43] comparing LCBDE during LC versus postoperative endoscopic retrograde cholangiopancreatography showed no significant difference in the success and complication rates between the two approaches; however, there is a need for fewer procedures and improved cost-effectiveness, as well as shorter hospital stay.

Conventional management of CBD stones included open laparotomy and bile duct exploration, which were first performed by Courvoisier and Thomton separately in 1889 [44]. Classic open CBD exploration is a proven safe and effective option for choledocholithiasis. With advances in laparoscopic technology, LCBDE can now be performed with efficiency and safety [45, 46]. A prospective randomized trial showed the mean operative time in the LCBDE group was similar to that in the laparotomy group, while the morbidity rate and length of postoperative hospitalization were lower in the LCBDE than in the open surgery group [22, 47]. Additionally, LCBDE can be performed without increasing the risk of bile duct complications [48]. Consistent with the modern concept of enhanced recovery after surgery, an LCBDE that exceeds the recommended time interval with no progress should be converted into other treatment options.

LCBDE is substantially better compared to endoscopic and open surgery with regard to hospital stay, postoperative pain, and cosmesis. LCBDE was first reported in 1991, and has been performed in combination with new technologies. It is considered safe and efficient [7, 8]. However, LCBDE has especially high technical requirements and may involve extensive manipulation of instruments such as balloon dilators, guide wires, catheters, and baskets, as well as laparoscopic suturing of the CBD. An argument against LCBDE is the potential risk of bile duct injury and strictures. With improved understanding of biliary anatomy and to reduce complications, some surgeons have attempted removal of CBD stones through the cystic duct instead of performing a direct incision over the CBD. The transcystic duct approach using the lumen of the cystic duct avoids the need to open the CBD; therefore, it may be preferred over a choledochotomy incision. As confirmed by our results, this would greatly reduce the occurrence of complications, especially biliary stricture and bile leakage. We observed that the LTCBDE group showed significantly lower biliary morbidity than the LCCBDE group. A T-tube acts as a foreign body around which bile pigments and salts can precipitate [16]. Therefore, in LTCBDE, the recurrence of stone formation was lower because it eliminated the subsequent need for a T-tube. Additionally, the complications caused by T-tube drainage, including water and electrolyte balance disorders caused by the loss of a large amount of bile, acid-base balance disorders, digestive dysfunction, retrograde infection, prolapse displacement, and living inconvenience were prevented. In fact, suturing of the cystic duct stump could be replaced with a knot or an absorbable clip. The trauma associated with LTCBDE is almost the same as that associated with LC; however, the advantage of LTCBDE is the minimal likelihood of an increase in complications. Although LTCBDE has many advantages, it also has limitations. Most importantly, complete removal of the stone is not always possible. A success rate of about 85% has been reported among all patients for LTCBDE [49]. The success of the transcystic approach depends on whether the choledochoscope is able to enter the CBD. The operation is relatively complex; hence, surgical expertise and adequate equipment (for lavage, biliary endoscopy, cystic duct dilation, trolling with wire baskets or balloon catheters, gravel lavage, and suturing) are indispensable. Moreover, the success of LTCBDE also depends on the anatomy of the cystic duct (duct diameter, and the bifurcation angle of the cystic and hepatic ducts) and CBD stones (location, size, and number of stones). Nevertheless, our results showed there was no significantl difference in operative time between the two groups.

The cystic duct connects the gallbladder and the bile duct. Its length is generally 3 cm and diameter is 0.2–0.3 cm. It consists of two parts, which including 5–12 consecutive half-moon mucosal folds called the Heister spiral valves and a smooth portion close to the CBD. Its elasticity and the smoothness of its interior are similar to the CBD. The muscles of the spiral folds are arranged like an annular valve, which can drive the bile flow by contraction and relaxation. Furthermore, the cystic duct itself functions like a sphincter and can coordinate gallbladder filling. The diameter of the confluence between the cystic and hepatic ducts is wider than the diameter of the CBD. The diameter of the cystic duct can expand to 1 cm or more when the CBD is obstructed, and the expansion is more obvious at the confluence. The anatomical features of the cystic duct and CBD create favorable conditions for LTCBDE.

The advantage of LTCBDE is that the integrity of the CBD is protected. Less injury to the CBD results in fewer postoperative complications. This explains the reduced length of hospital stay and lower hospital expenses for LTCBDE. For a large stone, a microincision at the enlarged confluence between the cystic duct and CBD can be performed; the diameter of the CBD has no significant effect-on the extent of suturing. Our results also showed that biliary morbidity, hospital expenses, and hospital stay in LTCBDE were significantly decreased, compared with LCCBDE. However, there was no significant difference in operative time between the two groups, which could be attributed to advances in suturing techniques. Previously, interrupted suturing was performed; however, suturing speed has greatly improved and the operative time has reduced by using barbed line or continuous sutures.

Preoperative magnetic resonance cholangiopancreatography can not only reveal the size and number of stones but also any variations in the biliary tract. LTCBDE can fail if the angle between the cystic and hepatic duct is small. Pulling the mucosa from the confluence of the cystic duct and CBD using an electrocautery hook and cutting it with scissors can reduce the failure rate caused by the small angle. However, if the incision along the cystic duct towards the CBD is too long, the advantage of using the LTCBDE approach is lost.

The results of our study should be interpreted with caution due to limitations. First, LTCBDE and LCCBDE utilize different levels of technology. Second, although we tried to identify all relevant data, potential publication bias was unavoidable and some data could have been missed (high-quality studies could have been excluded because of missing data or because the standards for LTCBDE were different for different teams). Finally, since this study was restricted to reports published in Chinese and English, publication bias could not be completely ruled out.

Although many experts have suggested criteria for LTCBDE, we believe that there are no fixed standards. The standards should be different for different teams, because they are affected by differences in the level of technology and the anatomical conditions. Based on our surgical experience, we have also developed corresponding standards.

LTCBDE provides a new understanding of biliary anatomy and the concept of choledocholithiasis treatment. Furthermore, it is a successful extension of laparoscopic biliary surgery technology. Although a longer learning time and more laparoscopic skills are required, it should be performed because of its good clinical efficacy.

## Supporting Information

S1 PRISMA Checklist (DOC)
