# Supplementary material for: Laparoscopic Transcystic Common Bile Duct Exploration: Advantages over Laparoscopic Choledochotomy
Source: PLoS One. 2016 Sep 26;11(9):e0162885. doi: 10.1371/journal.pone.0162885 (PMC5036868; doi:10.1371/journal.pone.0162885)
Supplement: S1 PRISMA Checklist — (DOC) [file pone.0162885.s001.doc]

| **Section/topic** | **#** | **Checklist item** | **Reported on page #** |
| --- | --- | --- | --- |
| **TITLE** | | |  |
| Title | 1 | **Laparoscopic transcystic common bile duct exploration: Advantages over laparoscopic choledochotomy** | Title  1 |
| **ABSTRACT** | | |  |
| Structured summary | 2 | *Purpose:* The ideal treatment for choledocholithiasis should be simple, readily available, reliable, minimally invasive and cost-effective for patients. We performed this study to compare the benefits and drawbacks of different laparoscopic approaches (transcystic and choledochotomy) for removal of common bile duct stones.  *Methods:* A systematic search was implemented for relevant literature using Cochrane, PubMed, Ovid Medline, EMBASE and Wanfang databases. Both the fixed-effects and random-effects models were used to calculate the odds ratio (OR) or the mean difference (MD) with 95% confidence interval (CI) for this study.  *Results:* The meta-analysis included 18 trials involving 2,782 patients. There were no statistically significant differences between laparoscopic choledochotomy for common bile duct exploration (LCCBDE) (*n*=1,222) and laparoscopic transcystic common bile duct exploration (LTCBDE) (*n*=1,560) regarding stone clearance (OR 0.73, 95% CI 0.50–1.07; *P*=0.11), conversion to other procedures (OR 0.62, 95% CI 0.21–1.79; *P*=0.38), total morbidity (OR 1.65, 95% CI 0.92–2.96; *P*=0.09), operative time (MD 12.34, 95% CI −0.10–24.78; *P*=0.05), and blood loss (MD 1.95, 95% CI −9.56–13.46; *P*=0.74). However, the LTCBDE group showed significantly better results for biliary morbidity (OR 4.25, 95% CI 2.30–7.85; *P*<0.001), hospital stay (MD 2.52, 95% CI 1.29–3.75; *P*<0.001), and hospital expenses (MD 0.30, 95% CI 0.23–0.37; *P*<0.001) than the LCCBDE group.  *Conclusions:* LTCBDE is safer than LCCBDE, and is the ideal treatment for common bile duct stones. | Abstract  2 |
| **INTRODUCTION** | | |  |
| Rationale | 3 | Approximately 10% of gallstone patients have concomitant common bile duct (CBD) stones [1, 2], which are related to serious complications such as cholangitis and pancreatitis. Therefore, it is particularly important to improve and standardize the process of diagnosis and treatment of CBD stones. The conventional approach of open CBD exploration is considered an effective treatment option [3-5]. However, surgical trauma, bile leakage, biliary tract blood loss, and other complications are not conducive to postoperative rehabilitation [6]. With rapid developments in technology, laparoscopic CBD exploration (LCBDE) was proven to be safe, cost-effective, and reliable, regardless of whether it was performed as elective or emergency treatment [7, 8]. Clinical prospective randomized trials have shown that laparoscopic procedures are superior to open operations with regard to reduced postoperative hospital stay, morbidity and postsurgical pain [9, 10]. | Introduction  3 |
| Objectives | 4 | The aim of this study was to pool analysis by investigating published data on LCBDE by the transcystic approach and choledochotomy. We assumed that LTCBDE is the ideal treatment for CBD stones. | Introduction  4 |
| **METHODS** | | |  |
| Protocol and registration | 5 | Indicate if a review protocol exists, if and where it can be accessed (e.g., Web address), and, if available, provide registration information including registration number. | No |
| Eligibility criteria | 6 | Case-control and cohort studies, published in English and Chinese only. | Material and methods  4 |
| Information sources | 7 | A systematic search was carried out using Cochrane, PubMed, Ovid Medline, EMBASE and Wanfang databases using the following keywords: bile duct exploration, laparoscopic CBD exploration, laparoscopic cholecystectomy, CBD stones, LCBDE, choledochotomy, laparoscopic transcystic CBD exploration, or LTCBDE. The latest search was updated on May 20th, 2016. | Material and methods  4 |
| Search | 8 | bile duct exploration, laparoscopic CBD exploration, laparoscopic cholecystectomy, CBD stones, LCBDE, choledochotomy, laparoscopic transcystic CBD exploration, or LTCBDE. | Material and methods  4 |
| Study selection | 9 | All controlled experimental studies about LTCBDE + laparoscopic cholecystectomy (LC) compared with LCCBDE+ LC were selected. The following were the inclusion criteria for the selected studies: (1) Patients with no contraindications for the laparoscopic approach, with no requirement of additional procedures; (2) Patients with confirmed or suspected CBD stones with gallstones; (3) Studies that included data on stone clearance, conversion to other procedures, total and biliary morbidity, blood loss (ml), length of hospital stay (days), operative time (min) and hospital expenses (wan renminbi). | Material and methods  4 |
| Data collection process | 10 | Data extraction was conducted independently by 2 investigators. Disagreement on article inclusion between the two reviewers was resolved via a third reviewer. | Material and methods  4 |
| Data items | 11 | The total morbidity (bile leakage, biliary stricture, bleeding, clinical pancreatitis, pneumonia, acute myocardial infarction, cholangitis, sepsis, cerebrovascular events, early reoperation, and pulmonary embolus), bile duct clearance and biliary morbidity (bile leakage and biliary stricture), conversion to other procedures (any endoscopic or surgical procedure other than the one allocated for failed bile duct clearance or any procedure for the management of a complication), operative time, length of hospital stay, hospital expenses, and blood loss were included. | Material and methods  5 |
| Risk of bias in individual studies | 12 | We used funnel plots to assess the publication bias, and tested for funnel plot asymmetry using Egger’s test and Begg’s test. | Material and methods  4 |
| Summary measures | 13 | Hazard Ratio | Material and methods  5 |
| Synthesis of results | 14 | The analyses were performed using Review Manager version 5.1 (RevMan, Cochrane Collaboration, Oxford, England). The results of this study were expressed as the odds ratios (ORs) for dichotomous data and mean difference (MD) for continuous data, with 95% confidence intervals (CIs) for both. The inverse variance method was used for continuous variables, while the Mantel-Haenzsel method was used for dichotomous variables. Statistical heterogeneity was evaluated by χ^2^ test. *P* < 0.05 was considered significant. If heterogeneity was significant, we used the random-effects model. Otherwise, we used the fixed-effects model. If data reported a median and range rather than a mean and standard deviation (SD), then the mean and SD were estimated as described previously [18]. | Material and methods  5 |

Page 1 of 2

| **Section/topic** | **#** | **Checklist item** | **Reported on page #** |
| --- | --- | --- | --- |
| Risk of bias across studies | 15 | The potential publication bias was evaluated by funnel plots. | Material and methods  5 |
| Additional analyses | 16 | Describe methods of additional analyses (e.g., sensitivity or subgroup analyses, meta-regression), if done, indicating which were pre-specified. | No |
| **RESULTS** | | |  |
| Study selection | 17 | See Fig 1. | Results  5 |
| Study characteristics | 18 | See table 1 | Results  5 |
| Risk of bias within studies | 19 | See Fig 1, Fig 2 and Fig 3, 4. | Results  5-6 |
| Results of individual studies | 20 | The meta-analysis included 18 trials involving 2,782 patients. There were no statistically significant differences between laparoscopic choledochotomy for common bile duct exploration (LCCBDE) (*n*=1,222) and laparoscopic transcystic common bile duct exploration (LTCBDE) (*n*=1,560) regarding stone clearance (OR 0.73, 95% CI 0.50–1.07; *P*=0.11), conversion to other procedures (OR 0.62, 95% CI 0.21–1.79; *P*=0.38), total morbidity (OR 1.65, 95% CI 0.92–2.96; *P*=0.09), operative time (MD 12.34, 95% CI −0.10–24.78; *P*=0.05), and blood loss (MD 1.95, 95% CI −9.56–13.46; *P*=0.74). However, the LTCBDE group showed significantly better results for biliary morbidity (OR 4.25, 95% CI 2.30–7.85; *P*<0.001), hospital stay (MD 2.52, 95% CI 1.29–3.75; *P*<0.001), and hospital expenses (MD 0.30, 95% CI 0.23–0.37; *P*<0.001) than the LCCBDE group. | Results  5-6 |
| Synthesis of results | 21 | **Study selection and characteristics**  Figure 1 illustrates the final selection of relevant studies and our search process. We analyzed 18 trials [19-36] that met the criteria, involving 2,782 patients (Table 1).  **CBD Stone clearance**  Stone clearance was reported in 12 trials. Stone clearance from the CBD was achieved in 87.3% of patients (693 of 794) in the LCCBDE group and in 88.9% (1,158 of 1,303) in the LTCBDE group. There was no significant difference between the two groups (OR 0.73, 95% CI 0.50–1.07; *P*=0.11) (Figure 2A).  **Conversion**  We identified ten trials with relevant data. Conversion occurred in 7.5% (62 of 831) and 10.9% (105 of 959) of patients in the LCCBDE and LTCBDE groups, respectively. There was also no statistically significant difference between the two groups (OR 0.62, 95% CI 0.21–1.79; *P*=0.38) (Figure 2B).  **Total** **morbidity**  Eleven trials reported total morbidity, with rates of 15.0% (117 of 781) and 10.3% (115 of 1,116) in the LCCBDE and LTCBDE groups, respectively. There was also no significant difference between the two groups (OR 1.65, 95% CI 0.92–2.96; *P*=0.09) (Figure 2C).    **Biliary morbidity**  Biliary morbidity was reported in nine trials, and occurred in 6.1% (52 of 855) of patients in the LCCBDE group versus 1.3% (13 of 1,000) in the LTCBDE group. The biliary morbidity for LTCBDE was significantly lower than in the LCCBDE group (OR 4.25, 95% CI 2.30–7.85; *P*<0.001). (Figure 2D).  **Operative time**  Twelve trials included data about for operative time. There was still no significant difference between the two groups (MD 12.34, 95% CI −0.10 to 24.78; *P*=0.05) (Figure 3A).  **Length of hospital stay**  The length of hospital stay was evaluated in 14 studies. Consequently, according to our predefined plan, the median and range were converted to mean and SD as described previously [18]. The length of hospital stay of the LTCBDE group was 2.52 days shorter than that of the LCCBDE group (MD 2.52 days, 95% CI 1.29–3.75; *P*<0.001) (Figure 3B).  **Hospitalization expenses**  The hospitalization charges were recorded in only three trials. The hospital expenses in the LTCBDE group were significantly lower than in the LCCBDE group (MD 0.30 WanRMB, 95% CI 0.23–0.37; *P*<0.001) (Figure 3E).  **Blood loss**  Only two trials included information about blood loss. There was still no significant difference between the two groups (MD 1.95 ml, 95% CI −9.56 to 13.46; *P*=0.74) (Figure 3D). | Results  6 |
| Risk of bias across studies | 22 | In this meta-analysis, the funnel plot shapes for postoperative complications and postoperative biliary complications showed basic symmetry (Figure 4). No significant publication bias was observed. The results were similar and the combined results were highly reliable. | Results  7 |
| Additional analysis | 23 | Give results of additional analyses, if done (e.g., sensitivity or subgroup analyses, meta-regression [see Item 16]). | No |
| **DISCUSSION** | | |  |
| Summary of evidence | 24 | The cystic duct connects the gallbladder and the bile duct. Its length is generally 3 cm and diameter is 0.2–0.3 cm. It consists of two parts, which including 5–12 consecutive half-moon mucosal folds called the Heister spiral valves and a smooth portion close to the CBD. Its elasticity and the smoothness of its interior are similar to the CBD. The muscles of the spiral folds are arranged like an annular valve, which can drive the bile flow by contraction and relaxation. Furthermore, the cystic duct itself functions like a sphincter and can coordinate gallbladder filling. The diameter of the confluence between the cystic and hepatic ducts is wider than the diameter of the CBD. The diameter of the cystic duct can expand to 1 cm or more when the CBD is obstructed, and the expansion is more obvious at the confluence. The anatomical features of the cystic duct and CBD create favorable conditions for LTCBDE. | Discussion  9-10 |
| Limitations | 25 | The results of our study should be interpreted with caution due to limitations. First, LTCBDE and LCCBDE utilize different levels of technology. Second, although we tried to identify all relevant data, potential publication bias was unavoidable and some data could have been missed (high-quality studies could have been excluded because of missing data or because the standards for LTCBDE were different for different teams). Finally, since this study was restricted to reports published in Chinese and English, publication bias could not be completely ruled out. | Discussion  11 |
| Conclusions | 26 | LTCBDE is safer than LCCBDE, and is the ideal treatment for common bile duct stones. | Discussion  9-10 |
| **FUNDING** | | |  |
| Funding | 27 | This work was supported by Youth Science Fund of Jiangxi Provincial Science and Technology Department (No.20161BAB215252). | Acknowledgments  11 |

*From:*  Moher D, Liberati A, Tetzlaff J, Altman DG, The PRISMA Group (2009). Preferred Reporting Items for Systematic Reviews and Meta-Analyses: The PRISMA Statement. PLoS Med 6(6): e1000097. doi:10.1371/journal.pmed1000097

For more information, visit: **www.prisma-statement.org**.

Page 2 of 2
